# Prevalence and Associated Explanatory Factors for Augmented Renal Clearance in Early Sepsis: Single-Center, Retrospective PICU Cohort in China, 2022–2023

**DOI:** 10.1097/PCC.0000000000003727

**Published:** 2025-03-20

**Authors:** Lili Xu, Jiayue Xu, Haoyun Mao, Wen Qian, Zhushengying Ma, Yuru Zhang, Yueniu Zhu, Xiaodong Zhu, Yaya Xu

**Affiliations:** All authors: Department of Pediatric Critical Care Medicine, Xinhua Hospital, Affiliated to the Medical School of Shanghai Jiaotong University, Shanghai, China.

**Keywords:** acute kidney injury, augmented renal clearance, glomerular hyperfiltration, sepsis

## Abstract

**OBJECTIVES:**

We aimed to 1) evaluate the prevalence of augmented renal clearance (ARC) in pediatric sepsis patients; 2) analyze potential explanatory factors associated with ARC out of hemodynamic, oxygenation, and inflammatory parameters; and 3) assess ARC outcomes.

**DESIGN:**

Retrospective, single-center, cohort from January 2022 to June 2023.

**SETTING:**

PICU at a tertiary care hospital in China.

**PATIENTS:**

Children 28 days to 16 years of age admitted with sepsis defined using Phoenix Sepsis Criteria.

**INTERVENTIONS:**

None.

**MEASUREMENTS AND MAIN RESULTS:**

Among 69 patients, 34 (49.3%) were categorized as having ARC. Designation as having ARC, vs. not, was associated with being younger (median 2.4 vs. 7.2 years, *p* < 0.001), hemodynamic and intrarenal flow changes, and higher C-reactive protein levels (31.0 vs. 12.0 mg/L, *p* < 0.05). None of the 34 patients with ARC developed acute kidney injury, but 22 of 35 non-ARC patients did. ARC was associated with shorter PICU stays (median 7 vs. 11 days, *p* < 0.05). Univariate regression analyses identified fluid balance, cardiac function parameters, renal resistive index, and inflammatory markers as explanatory factors associated with ARC.

**CONCLUSIONS:**

In this retrospective cohort of pediatric sepsis patients admitted to the PICU, the prevalence of early-onset ARC is around 50%, and younger patients may be at risk. The presence of ARC is associated with hemodynamic and inflammatory responses. Taken together, more prospective work is needed, with an emphasis on drug-level targeting and a better understanding of interactions with intrarenal pathophysiology.

**RESEARCH IN CONTEXT**

- Augmented renal clearance (ARC) in early pediatric sepsis, potentially leads to subtherapeutic drug levels and prolonged hospital stay.
- In this retrospective study, we have reviewed a cohort of pediatric sepsis managed in a single PICU in China.
- Our aim was to look at associations between categorization as having ARC and changes in hemodynamic, oxygenation, and inflammatory markers.

**AT THE BEDSIDE**

- In our retrospective study of PICU admissions with sepsis, we have found that ARC occurs in close to half of the patients.
- The main patient characteristic associated with exhibiting ARC in early sepsis was younger age, but other associated explanatory factors were fluid-, hemodynamic-, and inflammation-related.
- Further work is needed in individualizing antibiotic dosing in patients exhibiting signs of ARC.

Augmented renal clearance (ARC) is a state of enhanced renal filtration that can lead to increased elimination of renally excreted drugs, potentially resulting in subtherapeutic plasma concentrations and treatment failure at standard doses (1–4). A scoping review of literature 2015–2021 indicates that the prevalence of ARC in the PICU ranges from 7.8% to 78%, often increasing within the first week of admission (5). The 25 pediatric studies included in the ARC scoping review covered topics, such as antibiotics, like vancomycin and meropenem, the potential mechanisms underlying early ARC in sepsis, and the influence of renal functional reserve. Since the scoping review, there have been a few articles on the topic of antibiotic pharmacokinetics and dynamics in critically ill children (2, 3, 6, 7).

Therefore, in this retrospective study, we have evaluated the prevalence of ARC in pediatric sepsis patients managed in our PICU, 2022–2023. We have focused on explanatory variables related to hemodynamics, oxygen metabolism, and inflammation. Furthermore, we have assessed the association between ARC and clinical outcome.

## METHODS

This retrospective study is a post hoc analysis of an observational dataset that was collected between January 2022 and June 2023. The Institutional Research Board (IRB) and Ethics Committee of Xinhua Hospital (affiliated with Shanghai Jiao Tong University School of Medicine) approved the original study called “Novel Biomarkers for Early Renal Injury in Children With Sepsis” in January 2022 (No. XHEC-C-2022 to 012 to 1), which is ongoing and registered with the Clinical Trials Registry (NCT06197828) in December 2023. The ethics approval for the current post hoc report focused on ARC and is covered by the original IRB approval. In this analysis, all research procedures were conducted in accordance with the ethical standards of the institutional committee on human experimentation and the 1975 Declaration of Helsinki, including its later amendments.

### Cohort Identification and Definitions

The inclusion criteria for XHEC-C-2022 to 012 to 1 were as follows: 1) age 28 days to 16 years; 2) PICU length of stay (LOS) > 48 hours; 3) indwelling urinary catheter with completed renal resistive index (RRI) monitoring; and 4) at least two serum creatinine (SCr) measurements postadmission, with hourly urine output recorded. The exclusion criteria were as follows: 1) history of chronic renal insufficiency; 2) prior renal surgery; 3) family history of kidney disease or personal history affecting renal function; 4) refusal to sign informed consent; 5) incomplete data or follow-up records; and 6) history of abnormal muscle mass (e.g., amputee, spinal muscular dystrophy).

In this post hoc analysis, we applied the 2024 Phoenix sepsis diagnostic criteria (8). In the renal evaluation, we used the most recent SCr prior to PICU admission as the baseline value for the next 7 days. If data were missing, we used the 2021 Chinese reference for children’s SCr, selecting the minimum value by age and sex (**Table S1**, http://links.lww.com/PCC/C612) (9). Estimated glomerular filtration rate (eGFR) was calculated using the Schwartz-Lyon formula (10–12): eGFR=K×height(cm)/SCr(mg/dL). For males older than 13 years, *K* = 0.413; others, *K* = 0.367. We categorized patients into ARC and non-ARC groups based on 2024 reference values with eGFR cutoff of 99 mL/min/1.73 m^2^ for children young or younger than 2 years, and 140 mL/min/1.73 m^2^ for those older than 2 years (6).

We further divided the ARC and non-ARC groups into acute kidney injury (AKI) and non-AKI categories. For patients 1–16 years old, AKI was identified using the 2012 Kidney Disease: Improving Global Outcomes (KDIGO) guidelines (13). For those under 1 year, a modified KDIGO definition was used (14, 15). In patients on diuretics, SCr levels were prioritized to assess kidney damage.

### Data Collection and Calculations

Data were collected retrospectively using the electronic medical record. Data from the first 24 hours of PICU admission were analyzed. Renal function indicators (SCr, eGFR, blood urea nitrogen, uric acid, and neutrophil gelatinase-associated lipocalin [NGAL]) were collected. Hemodynamic parameters included mean arterial pressure, fluid balance, vasoactive inotropic score (VIS) (16), and cardiac/renal ultrasonography. Fluid balance was determined by subtracting total outputs (i.e., urine, drainage, stool, insensible water loss) from total intake (i.e., IV, blood, oral/nasogastric fluids), with insensible loss calculated using the Holliday and Segar equation (17).

Patients were grouped by daily cumulative fluid balance: fluid-negative (< 0%), fluid-balanced (0–10%), and fluid-overloaded (> 10%) (18). RRI was derived from renal ultrasound using the Schnell formula: RRI = (PSV–EDV)/PSV, where PSV is the peak systolic velocity and EDV is the end-diastolic velocity obtained by Doppler ultrasound of intrarenal blood flow (19).

Oxygen metabolism parameters were calculated and included arterial oxygen content (CaO_2_), mixed venous oxygen content, oxygen delivery (DO_2_), oxygen consumption, and oxygen extraction ratio (O_2_ER) (20). Arterial blood gas analysis, using ABL800 FLEX (Radiometer Medical, Copenhagen, Denmark), recorded lactate concentration, arterial partial pressures of oxygen, and carbon dioxide within 24 hours of admission. Urinary samples were collected from the bladder catheter within 24 hours. Urinary indicators comprised lactate concentration, and urine partial pressures of oxygen and carbon dioxide (21).

Antibiotic usage and inflammatory markers, including WBC count, neutrophil-to-lymphocyte ratio, C-reactive protein (CRP), interleukin-8 (IL-8), IL-1β, IL-6, tumor necrosis factor-alpha (TNF-α), and IL-10, were compared between groups.

Other interventions during hospitalization, such as continuous renal replacement therapy (CRRT) and mechanical ventilation (MV), were documented. Additional recorded outcomes included the PICU and hospital LOS, total hospital expenses, and mortality.

### Statistics

The Shapiro-Wilk test was used to assess data normality. Normally distributed continuous variables were expressed as mean ± sd and compared between groups using the independent samples *t* test. Non-normally distributed continuous variables were presented as median and interquartile range (P25, P75) and compared using the Mann-Whitney *U* test. Categorical variables were expressed as proportions and compared using the chi-square test or Fisher exact test. Differences in proportions are presented as absolute percentage and 95% CI. Potential explanatory variables with a *p* value of less than 0.001 were included in a univariate logistic regression analysis to identify associations with ARC. No corrections were made for multiple comparisons. Survival was analyzed with Kaplan-Meier (KM) curves.

## RESULTS

We identified 75 patients meeting the inclusion criteria who had been enrolled in XHEC-C-2022-012-1. We excluded included six patients: two with renal tumors, one who died within 24 hours, and three with incomplete urinary data. Therefore, we analyzed data from 69 patients: 35 in the non-ARC group and 34 in the ARC group (**Table 1**). Of note, being designated as ARC, as opposed to non-ARC, was associated with younger age (median 2.4 vs. 7.2 years, *p* < 0.001), and with correspondingly lower height and weight. Also, being designated as non-ARC, as opposed to ARC, was associated with more severe conditions, higher pediatric critical illness score, and pediatric risk of mortality III scores. We failed to identify an association between ARC category (ARC vs. non-ARC) and need for support with MV (7/34 vs. 13/35; absolute difference 16.5% [95% CI, −4.9% to 35.9%]; *p* = 0.134) or CRRT (2/34 vs. 7/35; absolute difference 14.1% [95% CI −2.5% to 30.6%], *p* = 0.084). However, the point estimate and effect sizes should also be looked at for what has not been excluded.

**TABLE 1.** Baseline Characteristics

Characteristics	Non-ARC (*n* = 35)	ARC (*n* = 34)	Total (*n* = 69)
Sex			
Male, *n*	18/35	24/34	42/69
Female, *n*	17/35	10/34	27/69
Age (yr)	7.2 (3.5, 10.4)	2.4 (0.6, 6.3)	6.1 (3.6, 7.2)
Height (cm)	114.0 (100.0, 135.0)	89.0 (66.0, 117.0)	103.0 (100.0, 117.0)
Weight (kg)	21.0 (15.0, 34.0)	12.5 (9.0, 20.6)	16.6 (15.0, 20.6)
Primary infected site			
Respiratory, *n*	22/35	17/34	39/69
Urinary, *n*	0/35	3/34	3/69
Skin, *n*	3/35	3/34	6/69
Gastrointestinal, *n*	4/35	8/34	12/69
Blood, *n*	3/35	0/34	3/69
CNS, *n*	3/35	3 (8.8)	6/69
Pediatric critical illness score	94.0 (86.0, 96.0)	100.0 (96.0, 100.0)	96.0 (96.0, 100.0)
Pediatric risk of mortality Ⅲ score	3.0 (0, 8.0)	2.0 (0, 5.0)	3.0 (2.0, 5.0)
Mechanical ventilation, *n*	13/35	7/34	49/69
Continuous renal replacement therapy, *n*	7/35	2/34	20/69
Length of PICU stay (d)	8.0 (6.0, 15.0)	7.0 (6.0, 13.0)	7.0 (7.0 9.0)
Length of hospital stay (d)	19.0 (12.0, 25.0)	19.0 (11.0, 27.0)	19.0 (24.0, 17.0)
Total hospital expenses (Chinese Yuan)	92,520.8 (58,735.2, 119,141.9)	88,988.3 (41,436.0, 94,173.3)	88,988.3 (74,842.1, 92,520.7)
Death, *n*	3/35	6/34	9/69

ARC = augmented renal clearance.

### AKI and Renal Biomarkers

**Figure 1.**
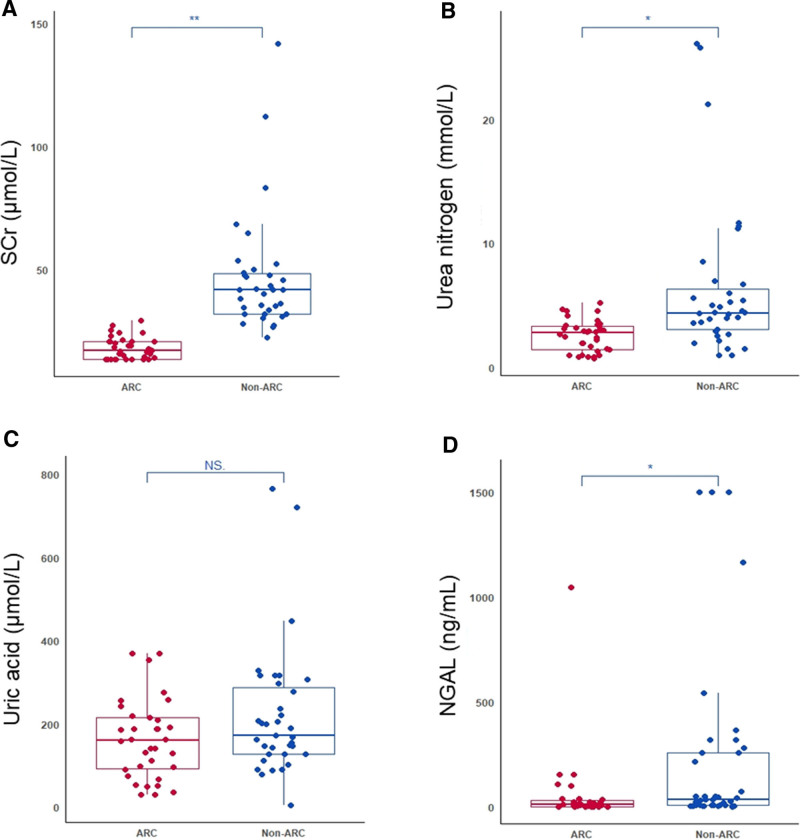
Comparison of renal function biomarkers between non-augmented renal clearance (ARC) and ARC groups: serum creatinine (SCr) comparison (**A**); urea nitrogen (**B**); uric acid (**C**); and neutrophil gelatinase–associated lipocalin (NGAL) (**D**). *Asterisks* denote statistically significant differences, *p < 0.05, **p < 0.001.

In the non-ARC group, 22 of 35 patients were diagnosed with AKI, whereas none of the 34 patients in the ARC group developed AKI. Being categorized as non-ARC, vs. being categorized as ARC, was associated with higher SCr (median 41.7 vs. 17.2 μmol/L, *p* < 0.001; **Fig. 1*A***), higher urea nitrogen (median 4.4 vs. 2.8 mmol/L, *p* < 0.05; **Fig. 1*B***), higher uric acid (median 171.4 vs. 159.8 μmol/L, *p* > 0.05; **Fig. 1*C***), and higher NGAL levels (median 37.9 vs. 10.8 ng/mL, *p* < 0.05, **Fig. 1*D***). Additionally, being categorized as non-ARC, as opposed to ARC, was associated with lower eGFR (101.7 vs. 161.3 mL/min/1.73 m², *p* < 0.001; **Fig. S1**, http://links.lww.com/PCC/C612).

### Hemodynamics, Oxygenation, and Inflammation

Comparison of hemodynamic data between ARC groups and non-ARC groups is shown in **Table S2** (http://links.lww.com/PCC/C612). Two of 34 patients designated as ARC daily cumulative fluid overload (i.e., > 10%); there were no other patients with fluid overload. Non-ARC designation was associated with higher VIS, mainly because of increased vasoactive drug use in the subgroup of non-ARC patients with AKI. We failed to identify an association between ARC grouping and cardiac function. However, regarding renal ultrasound measurements, ARC designation, as opposed to not, we did identify an apparent association (without correcting for multiple comparisons) with higher RRI, systolic/diastolic ratio, and pulsatility index. Even so, all of the values were within the normal accepted ranges, with no abnormal findings present (Table S2, http://links.lww.com/PCC/C612).

Regarding the oxygenation parameters, we failed to identify an association between ARC designation and changes in Cao_2_, DO_2_, and O_2_ER. Similarly, we failed to identify an association between ARC designation and differences in urinary and arterial blood gas analyses (Table S2, http://links.lww.com/PCC/C612).

The results of the associated differences in inflammatory markers, there was an association between ARC grouping and increased CRP levels, and 23/34 patients were receiving two or more antibiotics. We failed to identify any association between grouping with increased levels of other inflammatory markers (IL-8, IL-1β, IL-6, TNF-α) (Table S2, http://links.lww.com/PCC/C612).

### Outcome and ARC

**Figure 2.**
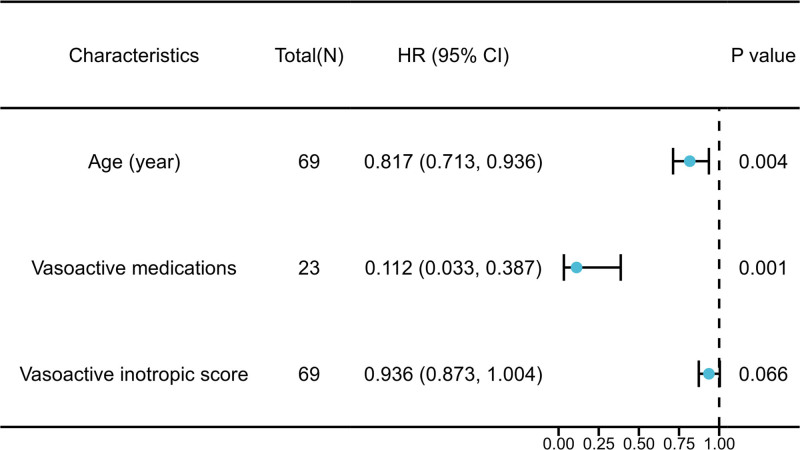
Univariate logistic regression analyses of factors associated with augmented renal clearance. HR = hazard ratio.

We further examined all variables in the exploratory bivariate analysis (i.e., with *p* < 0.001) in a univariate regression analysis. These explanatory variables included age; the use of vasoactive drugs; and the levels of VIS. The results show that older age and the use of vasoactive drugs may be associated with lower ARC prevalence, and modify any potential relationship (**Fig. 2**).

**Figure 3.**
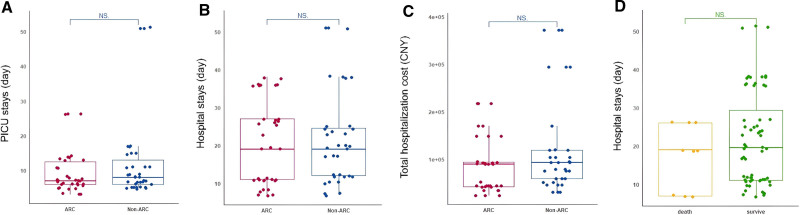
Comparison of clinical outcomes and costs between augmented renal clearance (ARC) and non-ARC groups. PICU stays in ARC and non-ARC groups (**A**); hospital stays in ARC and non-ARC groups (**B**); total hospital costs in ARC and non-ARC groups (**C**); and hospital stays in survivors and non-survivors (**D**).

**Figure 4.**
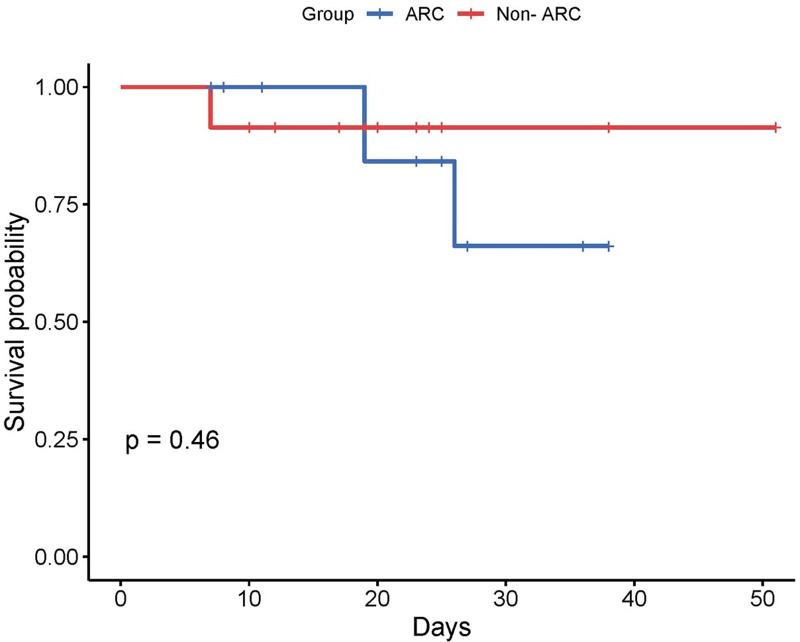
Kaplan-Meier curve analysis comparing survival probability between augmented renal clearance (ARC) and non-ARC groups.

The summary plots in **Figure 3** comparing ARC with non-ARC patients failed to show any associated relationship between grouping and PICU or hospital LOS, and costs. Overall, 9/69 patients died. We failed to identify an association between death, vs. survived, and overall hospital LOS (17.3 ± 3.3 vs. 21.8 ± 12.3 d; *p* = 0.334; **Fig. 3*D***). In the ARC group, 6/34 patients died with a mean hospital LOS of 22.5 ± 3.8 days, whereas in the non-ARC group, 3/35 patients died with a mean stay of 7.0 ± 0.1 days; there is an associated difference here, but the numbers are small, which likely reflect the time course of illness (**Fig. S2**, http://links.lww.com/PCC/C612). The KM survival curves are shown in **Figure 4**, which fail to demonstrate an association between ARC grouping and survival probability.

## DISCUSSION

In this retrospective study of a single-center 2022–2023 dataset of sepsis patients meeting Phoenix criteria, we have examined the prevalence of early ARC, which is 49.2% (95% CI, 37.0–61.6%) in our setting in China. In regard to potential explanatory factors out of hemodynamics, oxygenation, and inflammatory markers, the latter—with increased CRP—was the only variable robustly associated with ARC. Last, in regard to potential pathologic associations with ARC, the data point to vasopressors, and intrarenal blood flow physiology.

Our analysis shows that there is an association between sepsis complicated by ARC and being younger. Similar findings have been reported in a 2017–2022 cohort of 1453 critically ill children with hematological disease (22). The authors showed that ARC is associated with younger age and lower body mass index. Such an observation may be developmental. For example, in healthy infants and young children, renal clearance is dynamic, with the eGFR increasing in the first weeks and reaching adult levels by 2 years old. This maturation significantly impacts GFR in early life (6). However, some studies suggest that increasing age raises ARC risk, but it is unclear whether this phenomenon reflects how ARC is defined (23). For example, is ARC defined as eGFR ≥ 130 mL/min/1.73 m² without age adjustment, which potentially obscures the prevalence in younger children (23). Our study used SCr-based eGFR methods, with ARC determined by an eGFR cutoff of 99 mL/min/1.73 m² for children young or younger than 2 years, and 140 mL/min/1.73 m² for those older than 2 years, enhancing diagnostic sensitivity in younger patients (6). Currently, there is no standard or agreed definition and assessment for pediatric ARC. Some studies highlight that accuracy is highest with age- and sex-dependent eGFR equations (24). Therefore, individualized age-based calculations may be more suitable for diagnosing ARC in critically ill children.

In our renal function analysis, none of the patients in the ARC group developed AKI, likely because of both AKI and ARC diagnoses relying on SCr calculations, which complicates diagnosing AKI in ARC patients. Of note, designation by ARC (non-ARC vs. ARC) was associated with higher NGAL levels, which is a marker specific for kidney injury. Given that this feature is understudied, more work is needed. Here, for example, intrarenal flow dynamics and inflammation may be important. Historically, a more than 10-year-old study in critically ill adults with normal SCr concentrations suggests that ARC may result from compensatory responses to systemic inflammation and consequent altered renal function (25). A recent narrative review describes how, under the influence of cytokines, the kidneys may prioritize filtration over reabsorption, thereby leading to augmented clearance (26). To mitigate ARC, avoiding fluid overload and promptly addressing infection-induced inflammation aligns with sepsis treatment principles.

Studies published since the literature scoping review (5) indicate that ARC can reduce antibiotic trough concentrations, potentially extending PICU and hospital LOS (2, 3, 6, 7). In other sepsis-related conditions, ARC is also a problem. For example, in a retrospective analysis of a 2011–2021 cohort of 107 children with community-acquired bacterial meningitis, ARC was associated with prolonged antibiotic use, longer fever, and increased complications (27). Another retrospective study was an analysis of a 2012–2018 cohort of 32 neutropenic children with bacterial infection in the setting of malignancy (28). Here, the study found that 67% of meropenem and 60% of piperacillin levels were below target, with most samples from those with ARC. Taking the data together with the findings in our study, more research is needed to optimize and target blood antibiotic levels in patients with sepsis (4).

Our study does have limitations. It arises from a single center and has a small sample size that may affect generalizability outside of our local PICU setting. The retrospective design limits the interpretation of the results to assess associations and potential explanatory factors, which highlights the need for future prospective studies with larger cohorts. Additionally, we only used SCr levels at PICU admission to calculate renal function, without longer follow-up, which may limit the study’s scope. In the future, using multiple eGFR estimation methods could improve the diagnosis and assessment of ARC.

In conclusion, in this single-center retrospective study of patients admitted to our PICU with sepsis—defined using Phoenix criteria—in 2021–2022, we have found that the prevalence is around half of patients. We also found that younger age was associated with early-onset ARC. Last, ARC was also associated with intrarenal hemodynamics and systemic inflammation. Taken together, more prospective work is needed, with an emphasis on drug-level targeting and a better understanding of interactions with intrarenal pathophysiology.

## Supplementary Material


