# Supplementary material for: Prevalence and Associated Explanatory Factors for Augmented Renal Clearance in Early Sepsis: Single-Center, Retrospective PICU Cohort in China, 2022–2023
Source: Pediatr Crit Care Med. 2025 Mar 20;26(6):e788–95. doi: 10.1097/PCC.0000000000003727 (PMC12133050; doi:10.1097/PCC.0000000000003727)
Supplement: Supplementary file 1 [file pcc-26-e788-s001.docx]

**Supplemental materials**


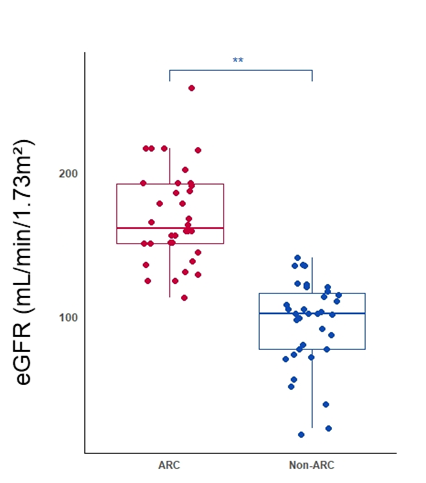


**Figure S1.** Comparative analysis of eGFR between Non-ARC and ARC Cohorts. ARC = augmented renal clearance; eGFR = estimated glomerular filtration rate. Asterisks denote statistically significant differences, ** *P*<0.001.


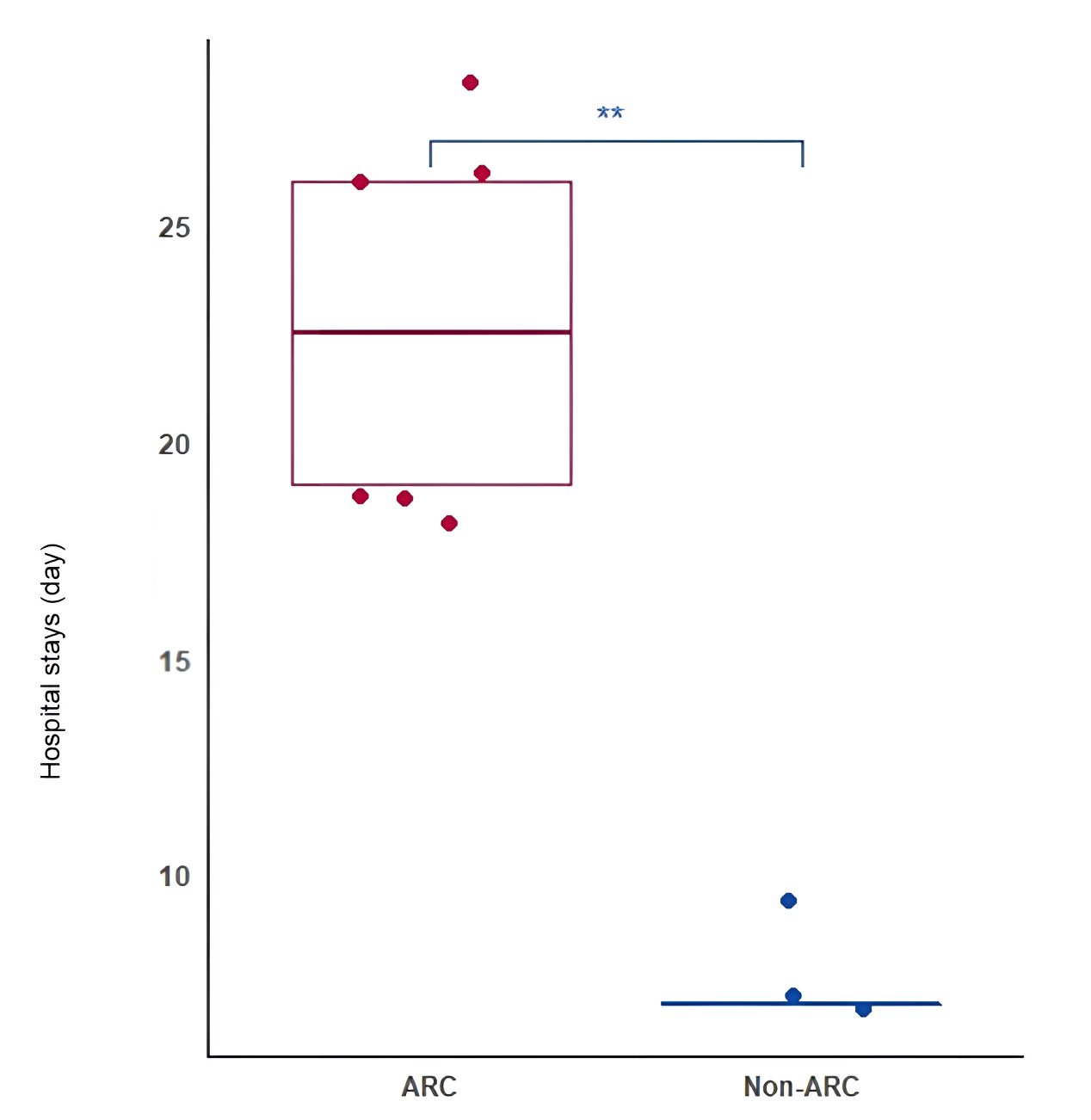


**Figure S2.** Comparison of hospital stays for deceased patients in ARC and Non-ARC groups. ARC = augmented renal clearance. Asterisks denote statistically significant differences, *** *P*<0.05, ** *P*<0.001

**Table S1 Reference Ranges for Common Biochemical Tests in Chinese Children**

| Item | Unit | Age | Reference Range | |
| --- | --- | --- | --- | --- |
|  |  |  | Male | Female |
| serum creatinine | μmol/L | 28 days~<2 years | 13～33 | |
|  |  | 2~ <6 years | 19～44 | |
|  |  | 6~<13 years | 27～66 | |
|  |  | 13~<16 years | 37～93 | 33～75 |
|  |  | 16~18 years | 52～101 | 39～76 |

**Table S2 Comparison of Hemodynamic, Oxygen Metabolism, and Inflammatory Indices Between Augmented Renal Clearance and Non-Augmented Renal Clearance Groups**

| **Clinical Metrics** | **Non-ARC (n=35)** | |  | **ARC (n=34)** | **Z/χ2 Value** | **P Value** |
| --- | --- | --- | --- | --- | --- | --- |
|  | **Non-AKI (n=13)** | **AKI (n=22)** |  | **Non-AKI (n=34)** |  |  |
| ***Hemodynamic Parameters*** |  |  |  |  |  |  |
| MAP (mmHg) | 73.7 (70.3, 82.3) | 81.8 (73.3, 97.0) |  | 76.2 (66.7, 82.3) | -1.333 | 0.183 |
| Vasoactive inotropic score | 0 | 9.0 (2.0, 15.0) ^a^ |  | 0 | -3.807 | <0.001 |
| Administration of vasoactive drugs, n | 1/13 | 18/22 ^a^ |  | 4/34 | 14.032 | <0.001 |
| Total of fluid intake and output (mL/kg) | 13.7 (11.5, 33.4) | 11.1 (2.1, 28.5) |  | 31.4 (9.2, 47.3) | 1.968 | 0.049 |
| ***Fluid balance*** |  |  |  |  |  |  |
| -fluid-negative, n | 1/13 | 7/22 |  | 4/34 | 2.924 | 0.225 |
| -fluid-balanced, n | 12/13 | 15/22 |  | 28/34 |  |  |
| -fluid-overloaded, n | 0/12 | 0/22 |  | 2/34 |  |  |
| ***Cardiac ultrasonography*** |  |  |  |  |  |  |
| -CO(L/min) | 4.1 (3.2, 4.9) | 4.1 (2.4, 6.3) |  | 2.5 (2.3, 4.6) | -1.999 | 0.046 |
| -EF (%) | 70.4 (63.4, 72.3) | 62.6 (45.4, 71.8) |  | 72.1 (68.8, 77.1) | 2.581 | 0.010 |
| -FS (%) | 38.3 (33.8, 41.1) | 33.1 (22.1, 40.5) |  | 39.6 (37.5, 43.6) | 2.233 | 0.026 |
| ***Renal ultrasound*** |  |  |  |  |  |  |
| -RRI (left kidney) | 0.6 (0.6, 0.7) | 0.6 (0.5, 0.6) |  | 0.6 (0.6, 0.7) | 2.061 | 0.039 |
| -S/D (left kidney) | 2.6 (2.3, 3.0) | 2.3 (2.0, 2.6) |  | 2.7 (2.4, 3.2) | 2.563 | 0.010 |
| -PI (left kidney) | 0.9 (0.8, 1.0) | 0.8 (0.7, 0.9) |  | 0.9 (0.8, 1.1) | 2.563 | 0.010 |
| ***Oxygen Metabolism-Related Parameters*** |  |  |  |  |  |  |
| CaO_2_ (mL O_2_ /dL) | 12.3 (11.1, 14.3) | 10.7 (8.9, 11.8) |  | 10.8 (8.9, 12.8) | -0.324 | 0.746 |
| CvO_2_ (mL O_2_ /dL) | 8.1 (6.2, 10.4) | 8.0 (6.8, 9.8) |  | 8.1 (7.1, 9.6) | -0.144 | 0.885 |
| DO_2_ (mL/min) | 450.8 (397.4, 603.5) | 434.3 (233.0, 744.8) |  | 293.6 (214.9, 554.6) | -1.752 | 0.080 |
| VO_2_ (mL O2/min) | 93.8 (63.5, 121.7) | 93.5 (37.8, 148.6) |  | 69.4 (32.5, 100.3) | -1.374 | 0.169 |
| O_2_ extraction ratio | 0.2 (0.2, 0.4) | 0.2 (0.1, 0.3) |  | 0.2 (0.2, 0.3) | 0.840 | 0.401 |
| PaO_2_ (mmHg) | 99.9 (74.4, 151.0) | 116.5 (51.2, 139.0) |  | 68.6 (43.4, 124.0) | -1.476 | 0.140 |
| PaCO_2_ (mmHg) | 37.8 (32.9, 41.4) | 41.7 (32.3, 52.3) |  | 38.4 (32.4, 42.7) | -1.068 | 0.285 |
| Blood lactic acid (mmol/L) | 1.2 (0.8, 2.1) | 1.3 (1.1, 2.3) |  | 1.1 (1.0, 1.9) | -0.902 | 0.367 |
| PuO_2_ (mmHg) | 148.0 (132.0, 154.0) | 149.0 (127.0, 161.0) |  | 149.0 (140.0, 155.0) | 0.312 | 0.755 |
| PuCO_2_ (mmHg) | 26.0 (21.8, 41.5) | 36.0 (17.9, 54.6) |  | 29.7 (18.6, 35.4) | -1.080 | 0.280 |
| Urine lactic acid (mmol/L) | 0.4 (0.2, 0.5) | 0.3 (0.2, 0.5) |  | 0.3 (0.2, 0.5) | -0.359 | 0.720 |
| **Inflammation-Related Data** |  |  |  |  |  |  |
| WBC (*10^9/L) | 7.0 (4.9, 9.2) | 7.3 (5.1, 12.0) |  | 7.6 (3.8, 9.8) | -0.036 | 0.971 |
| N/L | 4.9 (3.2, 5.9) | 6.3 (3.1, 15.1) |  | 1.5 (0.8, 6.9) | -2.761 | 0.006 |
| CRP (mg/L) | 24.0 (10.0, 69.0) | 36.0 (13.0, 73.0) |  | 58.0 (19.0, 519.0) | -3.007 | 0.003 |
| IL-8 (pg/mL) | 24.9 (23.6, 87.0) | 80.8 (23.6, 199.0) |  | 23.6 (23.6, 232.0) | -1.103 | 0.270 |
| IL-1β (pg/mL) | 5.0 (5.0, 6.1) | 5.0 (5.0, 35.6) |  | 7.8 (5.0, 9.4) | 0.680 | 0.496 |
| IL-6 (pg/mL) | 9.4 (5.8, 14.9) | 11.1 (5.8, 48.3) |  | 8.6 (5.8, 74.8) | 0.128 | 0.898 |
| TNF-α (pg/mL) | 14.9 (7.1, 21.8) | 10.3 (8.3, 50.9) |  | 13.4 (7.1, 19.7) | -0.743 | 0.458 |
| IL-10 (pg/mL) | 11.1 (9.7, 51.6) | 34.5 (9.7, 54.8) |  | 9.7 (6.7, 15.9) | -3.301 | 0.001 |
| Combination therapy with two or more antibiotics | 5/13 | 18/22 |  | 23/34 | 0.029 | 1.000 |
| ***Antibiotics*** |  |  |  |  |  |  |
| -β-lactam-β-lactamase inhibitors, n | 0/13 | 3/22 |  | 8/34 | 2.880 | 0.110 |
| -Cephamycins, n | 2/13 | 1/22 |  | 1/34 | 1.047 | 0.614 |
| -Glycopeptides, n | 5/13 | 7/22 |  | 19/34 | 3.251 | 0.092 |
| -Carbapenems, n | 4/13 | 14/22 |  | 13/34 | 1.213 | 0.336 |
| -Cephalosporins, n | 7/13 | 2/22 |  | 16/34 | 3.401 | 0.082 |
| -Aminoglycosides, n | 0/13 | 2/22 |  | 0/34 | 2.001 | 0.493 |
| -Oxazolidinones, n | 0/13 | 9/22 |  | 0/34 | 10.054 | 0.002 |
| -Penicillins, n | 0/13 | 2/22 |  | 0/34 | 2.001 | 0.493 |

MAP = mean arterial pressure, CO = cardiac output, EF = ejection fraction, FS = fractional shortening, RRI = renal resistive index, S/D = systolic/diastolic ratio, PI = pulsatility index, CaO_2_ = arterial oxygen content, CvO_2_ = venous oxygen content, DO_2_ = oxygen delivery, VO_2_ = oxygen consumption, PaO_2_ = arterial oxygen partial pressure, PaCO_2_ = arterial carbon dioxide partial pressure, PuO_2_ = pulmonary oxygen partial pressure, PuCO_2_ = pulmonary carbon dioxide partial pressure, WBC = white blood cell count, N/L = neutrophil-to-lymphocyte ratio, CRP = C-reactive protein, IL-8 = interleukin-8, IL-1β = interleukin-1 beta, IL-6 = interleukin-6, TNF-α = tumor necrosis factor alpha, IL-10 = interleukin-10, AKI=acute kidney injury, ARC= augmented renal clearance. Antibiotic usage in the study cohort, with some patients receiving combination therapy. Compared with Non-AKI group, ^a^*P* < 0.001.
